# Disordered Glucose Levels Are Associated with Xanthine Oxidase Activity in Overweight Type 2 Diabetic Women

**DOI:** 10.3390/ijms231911177

**Published:** 2022-09-23

**Authors:** Maria Elena Hernandez-Hernandez, Enrique Torres-Rasgado, Patricia Pulido-Perez, Leticia Nicolás-Toledo, Margarita Martínez-Gómez, Jorge Rodríguez-Antolín, Ricardo Pérez-Fuentes, Jose R. Romero

**Affiliations:** 1 Doctorate in Biological Sciences, Autonomous University of Tlaxcala, Tlaxcala 90070, Mexico; 2 Faculty of Medicine, Autonomous University of Puebla, Puebla 72420, Mexico; 3 Center for Biomedical Research East, Mexican Social Security Institute of Puebla, Atlixco 74360, Mexico; 4 Tlaxcala Center for Biology of Behavior, Autonomous University of Tlaxcala, Tlaxcala 90070, Mexico; 5 Department of Cellular Biology and Physiology, Biomedical Research Institute, National Autonomous University of Mexico, Mexico City 04510, Mexico; 6 Division of Endocrinology, Diabetes and Hypertension, Department of Medicine, Brigham and Women’s Hospital, Harvard Medical School, Boston, MA 02115, USA

**Keywords:** xanthine oxidase, overweight, obese, type 2 diabetic, biological sex

## Abstract

Oxidative stress plays an important role in vascular complications observed in patients with obesity and Type 2 Diabetes (T2D). Xanthine oxidase (XO) breaks down purine nucleotides into uric acid and contributes to the production of reactive oxygen species (ROS). However, the relationship between XO activity and glucose homeostasis in T2D subjects with obesity is unclear. We hypothesized that disordered glucose levels are associated with serum XO activity in overweight women and men with T2D and without hyperuricemia. We studied serum XO activity in women and men with and without T2D. Our results show that serum XO activity was greater in T2D patients with body mass index (BMI) ≥ 25 kg/m^2^ than in those with BMI < 25 kg/m^2^ (*p* < 0.0001). Sex-based comparative analyses of overweight T2D patients showed that serum XO activity correlated with homeostasis model assessment of β-cell function (HOMA-β), fasting plasma glucose (FPG), and hemoglobin A1C in overweight T2D women but not in overweight T2D men. In addition, as compared to overweight T2D men, women had higher high-sensitivity C-reactive protein (hs-CRP) levels. However, overweight T2D men had higher XO activity and uric acid levels than women. Our results suggest that XO activity is higher in overweight T2D patients, especially in men, but is more sensitive to disordered glucose levels in overweight women with T2D.

## 1. Introduction

Obesity is a risk factor contributing to the pathophysiology and onset of type 2 diabetes (T2D) through mechanisms that remain unclear [1]. Evidence shows that the prevalence of obesity in women and men with T2D is about 80–90% [2]. However, there is concern regarding biological sex differences that may affect the pathophysiology of obesity and T2D that remain unclear [3].

T2D and obesity are associated with an imbalance in the pro-oxidant/antioxidant system that promotes a state of chronic oxidative stress [4,5]. Overproduction of free radicals is mediated, in part, by increased plasma xanthine oxidase (XO) activity [6]. XO (EC 1.17.3.2) is the oxidant form of xanthine oxidoreductase (XOR), the enzyme that participates in the catabolism of purines to uric acid [7]. XO also can generate superoxide ions (O_2_^−^) and hydrogen peroxide (H_2_O_2_) [8,9,10]. XO activity is observed only in mammals [11]. In humans, XO activity has been shown in epithelial cells, liver, intestinal tissue, and breast tissue during lactation. Serum XO normally exists in very low concentrations, mainly due to hepatic cell turnover [7]. However, plasma XO levels and activity are increased under hypoxic/ischemic conditions and pathological conditions such as cardiovascular diseases and proinflammatory states [12].

There is evidence that XO contributes to the increased oxidative stress observed in T2D. Preclinical data shows that plasma malondialdehyde (MDA) levels, glutathione redox ratio, and liver protein oxidation in diabetic rats were higher than in non-diabetic rats. These markers of oxidative stress were decreased following treatment with allopurinol, an XO inhibitor [13]. Studies in humans have shown that serum XO activity is increased in patients with T2D [14,15]. In addition, plasma XO activity correlated with insulin resistance (IR) [16]. In addition, patients with the metabolic syndrome showed increased serum XO activity that correlated with body mass index (BMI) [17]. Consistent with these reports in humans, studies in liver and plasma from streptozotocin-induced diabetic Wistar rats showed higher XO activity when compared to rats without diabetes [18]. Others have reported that non-obese, non-hypertensive diabetic Goto-Kakizaki rats have increased hepatic XO activity in male vs. female rats [19]. However, whether the relationship between XO activity and glycemic control differs in men and women with T2D remains poorly understood as sex-disaggregated data and findings on sex-specific XO activity are not frequently reported. We hypothesized that disordered glucose levels would be associated with XO activity in overweight women and men with T2D.

## 2. Results

### 2.1. Characteristics of Study Population

In total, 227 subjects with and without T2D with normal levels of serum uric acid were included in this study (Table 1). A total of 92 men and 135 women between 30 to 65 years of age with a median age of 49.0 (41.0–57.0) years were studied. In our total population, 86 (37.9%) participants were overweight and 57 (25.1%) were obese. The metabolic evaluation showed that 98 (43.2%) participants were IR. Anthropometric and metabolic characteristics of our study subjects according to T2D status are presented in Table 1. Eighty-seven (38.3%) patients had T2D with a median of 5.0 (1.0–8.0) years of diagnosis. Comparative analyses between non-T2D subjects and T2D patients showed that a high proportion of patients with T2D were overweight (75.9%) and 64.4% were IR. Glycemic assessment showed that T2D patients had poor glycemic control, high C-peptide levels, lower β-cell function, and higher high-sensitivity C-reactive protein (hs-CRP) when compared to non-T2D subjects. XO activity in the T2D group was higher compared to those without T2D. An assessment of the pharmacological management of our T2D patients showed that 80.5% of the patients were treated with metformin and/or glibenclamide (50.6% metformin combined with glibenclamide, 23.0% with metformin alone, and 6.9% only with glibenclamide) and 19.5% of the patients in our study were untreated at the time of inclusion.

### 2.2. Serum XO Activity

**Figure 1:**
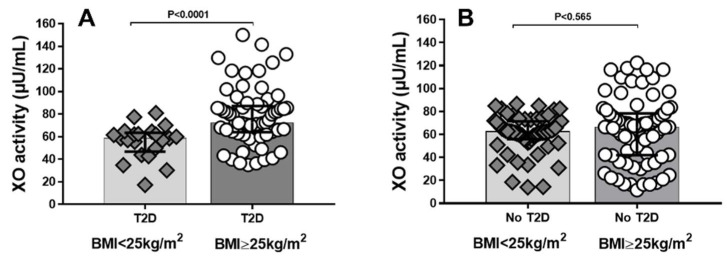
Xanthine oxidase (XO) activity in (**A**) patients with type 2 diabetes (T2D) grouped according to body mass index (BMI). (**B**) non-T2D (No-T2D). Data shown as median and interquartile range (IQR). Comparison between groups analyzed by Mann–Whitney U test. *p* < 0.05 was considered significant.

We evaluated serum XO activity in non-T2D and T2D patients grouped according to BMI. Figure 1A shows that T2D patients with BMI ≥ 25 kg/m^2^ had higher XO activity when compared to patients with T2D and BMI < 25 kg/m^2^ (*p* < 0.0001). We observed that among non-T2D subjects, there was no significant differences in XO activity when grouped according to BMI (*p* = 0.565) Figure 1B.

### 2.3. Sex-Based Analyses

Biological sex-based comparative analyses were performed on T2D patients and non-T2D subjects with BMI ≥ 25 kg/m^2^. Table 2 shows that no significant differences were observed in age, fasting plasma glucose (FPG), hemoglobin A1C (A1C), fasting plasma insulin (FPI), C-peptide, homeostasis model assessment of β-cell function (HOMA-β), and homeostasis model assessment for insulin resistance (HOMA-IR) between women and men in both T2D patients and non-T2D subjects. Uric acid levels were higher in men than in women with T2D and in non-T2D subjects. Serum XO activity was higher in T2D men than in women. However, as compared to men, hs-CRP was higher in T2D women than men. These differences were not observed in non-T2D overweight men and women. Age, FPG, A1C, HOMA-IR, and HOMA-β were higher in overweight T2D men and women versus overweight non-T2D men and women. C-Peptide was higher in overweight T2D patients as compared to non-T2D subjects as a shown in Table 2.

**Figure 2:**
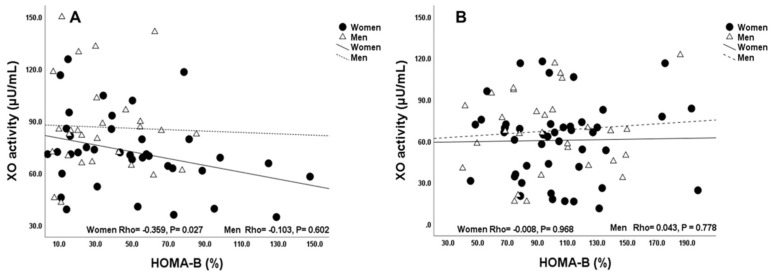
Association between serum XO activity and HOMA-β in overweight women and men with and without T2D. (**A**) T2D. (**B**) Non-T2D. The *p* values were estimated form Spearman’s correlation analyses. *p* < 0.05 was considered significant. Abbreviations: Xanthine oxidase (XO), Homeostatic Model Assessment of β-cell function (HOMA-β).

We performed sex-based correlation analyses of serum XO activity and measures of glycemic control in overweight T2D patients and non-T2D subjects. Our results show that serum XO activity was significantly correlated with HOMA-β in overweight T2D women (Rho = −0.359, *p* = 0.027) but not in T2D men (Rho = −103, *p* = 0.602) (Figure 2A). Overweight non-T2D subjects did not show any significant differences between women and men as shown in Figure 2B.

**Figure 3:**
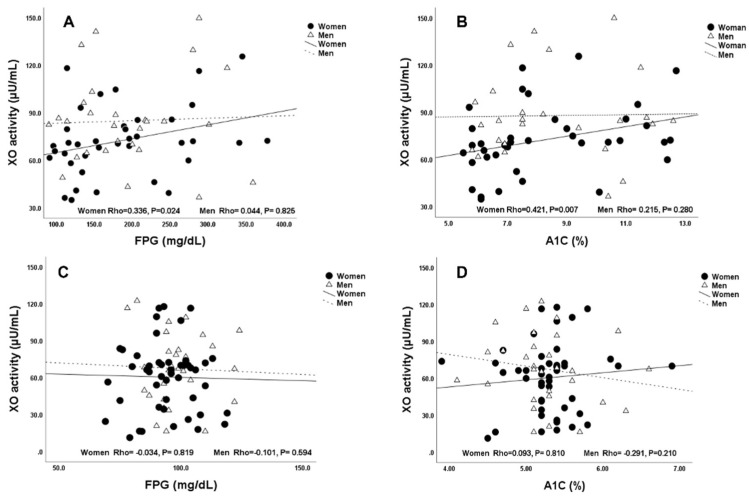
Correlation between XO activity and FPG and hemoglobin-A1C levels in T2D and non-T2D subjects with BMI ≥ 25 kg/m^2^. (**A**) XO activity vs. FPG in T2D patients. (**B**) XO activity vs. A1C in T2D subjects. (**C**) XO activity vs. FPG in non-T2D. (**D**) XO activity vs. A1C in non-T2D subjects. The P values were estimated by Spearman’s correlation analyses. Abbreviations: Xanthine oxidase (XO), fasting plasma glucose (FPG), hemoglobin-A1C (A1C).

Serum XO activity correlated with FPG (Rho = 0.336, *p* = 0.024) and % of A1C (Rho = 0.421, *p* = 0.007) in overweight T2D women but not in men as shown in Figure 3A,B. Correlations between XO activity and FPG and A1C parameters were not significant in both overweight non-T2D men and women as shown in Figure 3C,D.

## 3. Discussion

Our studies support the hypothesis that BMI and T2D contribute to increased XO activity in both women and men with normal uric acid. We show that XO activity correlates with measures of glucose homeostasis in women with T2D and altered BMI but not in men. These results suggest a novel role for glucose on XO activity that may be dependent on glycemic status, fat mass, and biological sex. These findings are of clinical importance as they add to the growing body of evidence that while men are at a higher risk of developing T2D [3], women with T2D have an increased risk of developing cardiovascular complications and increased mortality [20,21]. Furthermore, the risk of developing T2D complications in women increases at menopause [22].

Our results show that XO activity correlates with FPG and A1C in overweight T2D women. Consistent with these data, XO activity correlates with HOMA-β—a parameter that evaluates the function of the β-cell and its alteration is indicative of impaired glucose metabolism [23]. To the best of our knowledge, this is the first study documenting the relationship between XO activity and glycemic control in patients with T2D according to biological sex. These results suggests that glycemic control may play a key role in modulating XO activity. In addition, XO appears to be more sensitive to alterations in glucose metabolism in women than in men with T2D and obesity, thus implicating sex hormones in XO and reactive oxygen species (ROS) regulation. However, the reasons for these observations are not known. It is important to note that higher levels of oxidative stress markers have been reported in postmenopausal women as compared to women of reproductive age that is probably due to decreasing estrogen levels in the menopausal stage [24]. In addition, women, as compared to men, have a lower total antioxidant capacity, especially among postmenopausal women [25]. Of interest, in vitro studies show that estradiol treatment of human granulocytes is associated with lower anion superoxide (O_2_^−^) production compared to vehicle treated cells [26]. Additional studies are needed to clarify the mechanisms that can explain the association between parameters of glucose metabolism and XO activity in women. Of note and contrary to our results, a study in patients with T2D and hyperuricemia found no significant correlation between indices of glycemic control (A1C or FPG) and plasma XO activity [27]. However, this study did not assess the effects of biological sex on their outcomes [27].

We observed a significant difference in XO activity between overweight men and women with T2D. A literature review shows that XO activity and its relationship to biological sex is not clear. For example, a study of liver XO activity in patients without diabetes undergoing partial hepatectomy or open liver biopsy showed higher levels of XO activity in men than in women [28]. In contrast, others have shown the absence of any biological sex effects on enzyme activity [29,30]. Studies in wild-type rodents and rat models of T2D are more consistent. Sprague Dawley rats have higher liver XO activity in males than in female rats. Goto-Kakizaki rats (a model of T2D) also showed higher levels of hepatic XO activity in male than in female rats [19]. Although the precise mechanism(s) to explain the differences in XO activity between men and women is(are) unknown, some have proposed an effect of androgens [31] and estrogens [24]. It is important to note that uric acid is a product of XO activity, and its levels can be positively influenced by increased muscle mass as observed in men vs. women with T2D in our cohort.

There also is evidence to suggest that elevated XO activity may affect glucose metabolism. Studies show that treatment with allopurinol, a XO inhibitor, lowered A1C in T2D normotensive patients [32] and caused a decrease in markers of oxidative stress such as MDA in patients with T2D and mild hypertension [33]. However, a meta-analysis revealed that XO inhibition may have blood glucose-lowering effects only in individuals without T2D [34]. Consistent with our results, Kuppusamy et al. [15] showed a strong positive association between A1C and serum XO in patients with T2D suggesting that poor glycemic control is associated with increased XO activity.

Increases in adipose tissue also may independently contribute to greater XO activity. A small study in obese children reported an increase in XO activity compared to children with normal weight [35]. In obese adults, weight loss was associated with decreased XO activity [36]. Furthermore, there is evidence that XO may be key in regulating adipogenesis via peroxisome proliferator-activated receptor gamma (PPARγ) regulation that is essential in fat accumulation [37]. In fact, these investigators showed that in adipose tissue of obese mice, both the expression and activity of XO were increased. In line with these studies, there is evidence to suggest that human adipose tissue can overproduce the substrate hypoxanthine—an XO substrate that is secreted from human fat tissue especially under hypoxic conditions [38]. Consistent with these observations, obese subjects have been reported to have significantly higher serum hypoxanthine levels than non-obese subjects [39]. These findings suggest that increased hypoxanthine may contribute to elevated XO activity by a substrate-mediated increase in XO activation.

ROS produced by endothelial cells may compromise the integrity of the endothelium and lead to increased inflammatory responses and vascular damage in patients with T2D. Sex-based comparative analyses in patients with T2D and increased BMI showed that hs-CRP levels in women were higher than in men; raising the possibility that obesity and hyperglycemia may contribute to stimulating proinflammatory processes and ROS production in women. These alterations may, in turn, contribute to increased cardiovascular risk and worse outcomes in women [40]. Young women have a more favorable cardiovascular risk profile than men [41,42]. However, in women with T2D it would seem that this protection is lost [43,44]. Thus, we posit that in women, the presence of factors such as inflammation, obesity, and diabetes increase the sensitivity of XO to glucose.

Our study has some limitations. This is a cross-sectional study from one center. As such, we cannot provide a cause-and-effect relationship. First, the hormonal profile and reproductive status were not available in this study, limiting the analyses of potential impact of XO activity and menopausal status on parameters of glycemic assessment. Second, the age of the T2D group was higher compared to the non-T2D group, which may be a factor that additionally affects XO activity. Of importance and to the best of our knowledge, this study is the first to address the relationship between BMI, XO activity, and biological sex among patients with T2D. We provide information on the association between parameters of glycemic assessment and XO activity in T2D women and men. However, additional studies in larger populations that are age- and biologically sex-matched are needed to confirm our findings and prospectively assess the relationship between XO activity, glucose metabolism, and menopausal status. Our novel findings provide the rationale for the development of such studies.

## 4. Materials and Methods

### 4.1. Subjects and Settings

This cross-sectional study included 227 participants, men (*n* = 92) and nonpregnant women (*n* = 135) between the ages of 30 to 65 years of age. Eighty-seven subjects with previous medical diagnosis of T2D and 140 non-T2D were included in our study. The T2D patients are beneficiaries of the medical services of the Family Medical Unit Number 2 (UMF-2) of the Mexican Social Security Institute (IMSS) in Puebla, Mexico. For the recruitment of the non-T2D group, an open invitation was made for family members of T2D patients. All participants provided signed informed consent before study participation and were evaluated for a complete clinical and family history. Anthropometric and biochemical parameters were determined. Exclusion criteria included: incomplete clinical history or blood sampling, chronic proinflammatory diseases (arthritis, autoimmune disease, malignancy), endocrine diseases (hyperthyroidism, hypothyroidism, or Cushing’s disease), or if the subjects were receiving anti-inflammatory treatments. Subjects with hyperuricemia were excluded because elevated uric acid levels are associated with XO hyperactivity in patients with diabetes [45,46]. Patients with T2D and insulin therapy or >10 years of evolution of the disease also were excluded from the study. The study was approved by the IMSS National Ethics and Scientific Research Committee (R-2020-785-013).

### 4.2. Clinical and Anthropometric Characterization

Using standardized protocols, all participants were evaluated including their individual and family clinical history. Height (meters), weight (kg), and muscle mass (Kg) were measured using an electronic digital scale (Tanita Body Composition Analyzer, Model TBF-215, Tokyo, Japan), and BMI was calculated as weight/height^2^ (kg/m^2^). A BMI between 18.5–24.9 kg/m^2^ was considered normal, between 25–29.9 kg/m^2^ was considered overweight, and between 30–39.9 kg/m^2^ was obese [47]. BMIs < 18.5 kg/m^2^ and >40 kg/m^2^ were excluded from this study.

Venous blood was taken after an overnight fast (10–12 h) between 7 am–10 am by venipuncture. Blood samples were collected in K_2_EDTA to obtain whole blood or plasma and in a tube without anticoagulants to obtain serum. FPG and hemoglobin-A1C were determined according to conventional laboratory protocols (Clinical Analyzer System from Beckman Coulter, Indianapolis, IN, USA). FPI and C-peptide levels were determined by immunoassay utilizing anti-insulin or anti-C-peptide mouse monoclonal antibodies respectively with alkaline phosphatase (Roche E170 analyzer, Roche Diagnostics, Mannheim, Germany). β-cell function and IR were determined by the HOMA: HOMA-β = [20 × FPI (mU/L)]/[FPG (mmol/L) − 3.5] and HOMA-IR = [FPI (mU/L) × FPG (mmol/L)]/22.5. The HOMA-β was used to evaluate the β-cell function and is expressed as a percentage of normal (100%). The HOMA-IR is used to evaluate the index of IR [48]. IR was defined by a HOMA-IR >2.6 [49]. hs-CRP levels were determined in serum to assess the inflammatory status of the participants using an immunoturbidimetric assay (DiaSorin, Stillwater, MN, USA).

### 4.3. XO Activity

The XO activity was measured in serum containing samples using a fluorometric assay (Xanthine oxidase: Cayman, Ann Arbor, MI, USA, Item No. 10010895). The assay is based on a multistep enzymatic reaction in which XO first produces H_2_O_2_ during oxidation of hypoxanthine. In the presence of horseradish peroxidase, the H_2_O_2_ reacts with 10-acetyl-3,7-dihydroxyphenoxazine in 1:1 stoichiometry to produce resorufin, a fluorescent compound, which was measured with at an excitation wavelength of 520–550 nm and emission wavelength of 585–595 nm.

### 4.4. Allocation of Subjects into Groups and Subgroups

Glycemic assessment was done according to measures of FPG, FPI, C-peptide, and HOMA-β. Glycemic control was categorized using the recommendations of American Diabetes Association (ADA) and the Guideline for Diagnosis and Pharmacological Treatment of T2D. These included: FPG ≥ 126 mg/dL (7.0 mmol/L) or A1C ≥ 6.5 % (48 mmol/mol) [50]. These two groups were sub-divided by BMI: normal weight (BMI < 25 kg/m^2^) or overweight (BMI ≥ 25 kg/m^2^).

### 4.5. Statistical Analyses

Data were analyzed and presented using descriptive statistics. Continuous variables were expressed as a median and interquartile range, and categorical variables as frequencies and percentages. The Kolmogorov–Smirnov test was used to determine the normality of the data distribution. Comparisons between groups were tested using the Chi-square test for categorical variables, and the Mann–Whitney U test for continuous variables. Comparisons between subgroups were analyzed utilizing the Kruskal–Wallis test. A Spearman correlation test was used to investigate the association between serum XO activity with measures of glycemic evaluation (FPG, A1C, FPI, C-peptide, and HOMA-β and HOMA-IR). Differences and correlation analyses between non-diabetic and T2D patients were considered statistically significant for *p* < 0.05. BMI-based analyses for non-T2D subjects and T2D patients were corrected by the Bonferroni post hoc test for multiple comparisons and correlation analyses, using a significance threshold of 0.0125 (0.05/4 comparisons). Statistical analyses were performed using SPSS for Windows version 24.0 (SPSS, Chicago, IL, USA). Statistical charts were generated using GraphPad Prism for Windows version 7.0.0 (San Diego, CA, USA).

## 5. Conclusions

XO activity is higher in overweight T2D patients, especially in men, but is sensitive to disordered glycemic control in overweight women with T2D. These findings suggest that biological sex and measures of glucose homeostasis are biological variables that affect XO activity in patients with T2D. Future studies that assess the mechanisms by which glucose regulates ROS homeostasis and the contributions of XO activity and sex-hormones in women and men with and without T2D are needed. Understanding these mechanisms in women is of critical importance as they may assist in explaining pathophysiological mechanisms for cardiovascular disease in women with T2D and lead to the development of novel and/or more precise therapeutic approaches. The importance of these studies is further underscored by reports showing that women with T2D, as compared with men, have an increased risk of developing cardiovascular complications and increased mortality through mechanisms that are not entirely clear.

## Figures and Tables

**Table 1:** Clinical characteristics of the study participants according to T2D.

Parameter	Non-T2D (*n* = 140)	T2D (*n* = 87)	*p*
Men/women, *n* (%)	56 (40.0)/84 (60.0)	36 (41.4)/51 (58.6)	0.890
Age (years)	45.0 (39.0–54.0)	56.0 (46.0–61.0)	<0.0001
BMI (kg/m^2^)	25.4 (22.4–28.9)	28.3 (25.0–31.6)	<0.0001
FPG (mg/dL)	94.0 (87.2–102.0)	165.0 (127.0–229.0)	<0.0001
A1C (%)	5.1 (4.7–5.5)	7.5 (6.6–10.4)	<0.0001
FPI (mg/dL)	8.1 (5.6–12.3)	8.4 (5.7–13.0)	0.393
C-Peptide (mg/dL)	2.1 (1.6–2.7)	2.7 (2.1–3.3)	<0.0001
HOMA-IR	1.8 (1.3–2.7)	3.4 (2.3–6.6)	<0.0001
HOMA-β	102.6 (69.1–133.0)	33.6 (15.2–58.2)	<0.0001
Uric Acid (mg/dL)	5.0 (4.0–5.7)	4.8 (4.0–5.2)	0.231
hs-CRP (mg/dL)	1.3 (0.5–3.2)	1.8 (1.0–4.2)	0.028
XO (μU/mL)	63.5 (53.4–73.1)	70.7 (58.1–84.6)	0.009
BMI > 25 kg/m^2^, *n* (%)	77 (55.0)	66 (75.9)	0.001

Data shown as median and interquartile range (IQR). The comparison between the groups was carried out by the Mann–Whitney U test. *p* < 0.05 was considered significant. Abbreviations: body mass index (BMI), fasting plasma glucose (FPG), hemoglobin-A1C (A1C), fasting plasma insulin (FPI), homeostatic model assessment for Insulin resistance (HOMA-IR), homeostasis model assessment of β-cell function (HOMA-β), hs-CRP (high-sensitivity C-reactive protein), xanthine oxidase (XO), type 2 diabetes (T2D).

**Table 2:** Sex-based analyses of overweight T2D patients and non-T2D subjects.

Parameter	Overweight Non-T2D (77)		Overweight T2D (66)	
	Men (*n* = 30)	Women (*n*= 47)	Men (*n* = 28)	Women (*n*= 38)
Age (years)	41.0 (30.0–49.5)	45.0 (39.0–54.0)	58.5 (47.2–63.0) ^†^	57.0 (47.5–65.0) ^‡^
FPG (mg/dL)	95.0 (93.0–102.5)	94.0 (87.0–103.0)	179.5 (137.0–236.2) ^†^	168.0 (119.7–249.0) ^‡^
A1C (%)	5.2 (4.8–5.6)	5.3 (5.0–5.6)	7.5 (6.7–10.6) ^†^	7.5 (6.2–10.2) ^‡^
FPI (mg/dL)	10.1 (6.6–12.7)	8.8 (6.2–13.5)	8.5 (5.6–13.0)	10.7 (7.7–15.2)
C-Peptide (mg/dL)	2.3 (1.8–2.8)	2.3 (1.7–3.1)	3.0 (2.4–3.9) ^†^	2.7 (2.3–3.6)
HOMA-IR	1.3 (0.9–1.6)	1.2 (0.8–1.8)	4.1 (2.2–6.6) ^†^	5.0 (2.6–8.1) ^‡^
HOMA-β	93.9 (73.9–123.6)	98.8 (77.3–119.7)	29.2 (14.9–54.4) ^†^	46.0 (15.7–72.2) ^‡^
hs-CRP (mg/dL)	1.5 (0.7–3.0)	1.5 (0.5–4.1)	1.3 (0.7–3.2)	2.4 (1.5–4.5) ^§^
Uric Acid (mg/dL)	6.0 (5.0–6.1)	4.3 (4.0–5.0) ^Ŧ^	5.0 (4.0–6.0)	4.0 (3.9–5.0) ^§^
XO activity (μU/mL)	67.4 (44.8–87.8)	66.5 (36.2–72.5)	83.6 (67.5–94.8)	71.1 (61.2–82.6) ^§^

Data shown as median and interquartile range (IQR). The comparison between the groups was carried out by the Kruskal–Wallis test adjusted with the Bonferroni correction for repeated samples. Abbreviations: fasting plasma glucose (FPG), hemoglobin-A1C (A1C), fasting plasma insulin (FPI), homeostatic model assessment for Insulin resistance (HOMA-IR), homeostasis model assessment of β-cell function (HOMA-β), hs-CRP (high-sensitivity C-reactive protein), xanthine oxidase (XO), type 2 diabetes (T2D). ^§^ T2D Women vs. T2D Men. ^‡^ Non-T2D Women vs. T2D Women. ^†^ Non-T2D Men vs. T2D Men. ^Ŧ^ Non-T2D women vs. non-T2D men.

## Data Availability

The data that support the findings of this study are available upon request from the corresponding author following reasonable request and will be considered on a case-by-case basis from qualified researchers with approvals from the Ethics Committee, Institutional review board, and executed institutional data transfer agreements.
